# Prevalence of trigeminocervical convergence mechanisms in episodic and chronic migraine

**DOI:** 10.1590/0004-282X-ANP-2021-0095

**Published:** 2022-02-28

**Authors:** Marco Antonio Takashi Utiumi, João Guilherme Bochnia Küster, Keryn Sporh Godk, Maria Luiza dos Santos, Bin Cheng Tan, Eldislei Mioto, Nikolai José Eustátios Kotsifas, Luiz Carlos Canalli, Gabriel Eduardo Faria Colombani, Pedro André Kowacs, Elcio Juliato Piovesan

**Affiliations:** 1 Universidade Federal do Paraná, Hospital de Clínicas, Programa de Pós-Graduação em Medicina Interna, Departamento de Clínica Médica, Curitiba PR, Brazil.; 2 Clínica de Neurologia São José, São José dos Pinhais PR, Brazil.; 3 Hospital Marcelino Champagnat, Serviço de Neurologia, Curitiba PR, Brazil.; 4 Universidade Federal do Paraná, Curso de Medicina, Setor de Ciências da Saúde, Curitiba PR, Brazil.

**Keywords:** Headache, Migraine Disorders, Neck Pain, Cefaleia, Transtornos de Enxaqueca, Cervicalgia

## Abstract

**Background:**

Migraine pain location and trigeminocervical convergence have limited diagnostic value and have usually been assessed using non-standard verbal descriptors in a small number of centers.

**Objective:**

To use non-verbal descriptors of migraine pain location to determine the prevalence of trigeminocervical convergence mechanisms in patients with episodic and chronic migraine. In addition, we explored the factors associated with the presence of convergence.

**Methods:**

A multicenter study was carried out. The explicit pain location was explored by asking subjects to indicate, on an electronic form, three points on the anterolateral side and three points on the posterolateral side of the head and neck that represented the common locations of their migraine pain. We evaluated associations of the pain pattern with demographic and psychological features, comorbidities, lifestyle and other headache characteristics.

**Results:**

97 episodic and 113 chronic migraine patients were included. Convergence was present in 116 migraineurs (55%) who indicated dominance of pain in the posterior cervical region. This site was more often involved in the chronic migraine group (21 vs. 33%; p=0.034). The number of migrainous/altered sensitivity symptoms (OR=1.39; 95%CI 1.14–1.71) was associated with convergence independently of the chronification status. In this symptom group, there were statistical associations between convergence and vomiting (p=0.045), tactile allodynia (p<0.001), nuchal rigidity (p<0.001) and movement allodynia (p=0.031).

**Conclusions:**

Trigeminocervical convergence is common in migraineurs and, in practice, it might be found frequently in chronic migraineurs. Some features commonly found in this group, such as altered sensitivity symptoms, are associated with this phenomenon.

## RESUMO

**Antecedentes:**

A localização da dor da migrânea e a convergência trigeminocervical têm valor diagnóstico limitado geralmente sendo avaliadas por meio de descritores verbais não padronizados em um pequeno número de centros.

**Objetivo:**

Usar descritores não verbais da localização da dor migranosa para determinar a prevalência de mecanismos de convergência trigeminocervical em pacientes com migrânea episódica e crônica. Além disso, exploramos os fatores associados à presença de convergência.

**Métodos:**

Um estudo multicêntrico foi realizado. A localização explícita da dor foi explorada solicitando aos migranosos que indicassem, em um formulário eletrônico, três pontos no lado anterolateral e três pontos no lado posterolateral da cabeça e pescoço representando a localização comum de sua dor migranosa. Avaliamos a associação do padrão de dor com características demográficas e psicológicas, comorbidades, estilo de vida e outras características da cefaleia.

**Resultados:**

97 pacientes com migrânea episódica e 113 com migrânea crônica foram incluídos. A convergência esteve presente em 116 migranosos (55%) que indicaram um predomínio da dor na região cervical posterior. Este local estava mais frequentemente envolvido no grupo migrânea crônica (21 *vs*. 33%; p=0,034). O número de sintomas de migrânea/sensibilidade alterada (OR=1,39; IC95% 1,14–1,71) foi associado à convergência independentemente do estado de cronificação. Nesse grupo de sintomas, houve associações entre convergência e vômito (p=0,045), alodinia tátil (p<0,001), rigidez de nuca (p<0,001) e alodinia ao movimento (p=0,0031).

**Conclusões:**

A convergência trigeminocervical é comum em pacientes com migrânea e, na prática, podemos encontrá-la com frequência em pacientes com migrânea crônica. Algumas características comumente encontradas nesse grupo, como sintomas de sensibilidade alterada, estão associadas a esse fenômeno.

## INTRODUCTION

The diagnosis of migraine is based on well-defined clinical characteristics that are described in the International Classification of Headache Disorders, 3^rd^ edition (ICHD-3)^1^. A comment about the clinical expression of migraine in the ICHD-3 recognizes the frontotemporal region as the usual migraine pain site. However, other spatial information has no formal diagnostic value.

In the largest study (n=1,283) dedicated to the topic of migraine pain location, Kelman found that the orbital, frontal and temporal sites were the regions most prevalently affected by migraine^2^. Interestingly, this study highlighted the diagnostic value of the pain site by demonstrating that episodic migraine (EM) affects the eyes more frequently than does chronic migraine (CM), which, in turn, is characterized by neck, occipital and diffuse pain.

Most studies have used similar verbal descriptors to categorize the migraineurs’ pain location. However, use of terms like “diffuse” and “eyes” to describe unspecific or composite sites might hinder future comparisons. Also, the way in which borderline regions are reported could result in overestimation or underestimation of the involvement of some sites.

Fernándes-de-las-Peñas et al. demonstrated the use of drawings to explore pain extent in migraineurs^3^. More recently, Uthaikhup et al. used the drawings of 114 participants to compare pain location in EM (n=48), CM (n=30), and cervicogenic headache and explored the associations between location and other headache features, psychological distress and quality of life^4^. They found that the frontal and temporal regions were the most commonly affected sites in migraineurs, with a trend towards more posterior pain in the CM group. Larger pain extent was correlated with higher headache intensity and worse quality of life in CM.

These studies show an intriguing association between CM and more posterior (extratrigeminal sites) and/or diffuse pain.

Although migraine is predominantly associated with dysfunction of the trigeminal system, a large number of articles have demonstrated that activation of the high cervical nociceptive system occurs during attacks. This correctly reclassifies migraine as a pathological condition related to dysfunction of the trigeminocervical system. The caudal subnucleus of the trigeminal nucleus is an intermediate structure of the cranial nociceptive system that receives nociceptive afferents from the first and second branches of the trigeminal and the first cervical roots, resulting in a convergence mechanism known as trigeminocervical convergence^5^. This physiological phenomenon has been clinically demonstrated through chemical stimuli on the major occipital nerve, which generated immediate pain radiating to the first branch of the trigeminal tract, mainly in the orbital and supraorbital region^6^. On the other hand, an anesthetic block of the major occipital nerve may be able to control migraine attacks^7^.

Hence, migraine is not a disorder that is static in space; and not even in time and intensity. The episodic form (EM) can evolve to a progressive form (CM) characterized by increases in the frequency, severity, duration and refractoriness of the attacks. Genetic factors are probably involved in the neuronal mechanisms inside structures participating in pain processing^8^. However, the influence of these alterations on the trigeminocervical process is unclear.

The aim of the present study was to use non-verbal descriptors of migraine pain location to identify the prevalence of the involvement of extratrigeminal sites (a manifestation of trigeminocervical convergence) in the EM and CM groups. As a secondary objective, we explored the factors associated with the presence of convergence, including a wide range of variables related to demographic and psychological features, comorbidities, lifestyle and other migraine characteristics.

## METHODS

This research was originally designed to explore the role of certain genetic polymorphisms in the clinical expression of migraine. The present study is a clinical data analysis on the pain location pattern in our sample.

This study was approved by the Ethics Committee of Hospital de Clínicas, Universidade Federal do Paraná (registration 2.732.610; CAAE number: 87998518.8.0000.0096) and was registered in the Brazilian Registry of Clinical Trials (RBR-9wgwnj). Written consent was obtained from all the patients prior to data collection.

We used a case-control design to compare different patterns of migraine pain location between CM (cases) and EM (controls). Three centers participated in this research: one tertiary-level healthcare center that exclusively serves the public healthcare system (Hospital de Clínicas, Universidade Federal do Paraná); and two headache outpatient clinics (Clínica de Neurologia São José and Hospital Marcelino Champagnat).

All the subjects invited to participate in the study presented the following characteristics: (1) they had a definitive diagnosis of EM or CM (either associated with analgesic abuse or not), in accordance with the ICHD-3; (2) they had been suffering from migraine symptoms for at least six months before the research interview; (3) they had no limitations on provision of information; (4) they had no associated condition that could make the migraine diagnosis uncertain (e.g. HIV infection, active cancer or use of immunosuppressive drugs); (5) they were 18 years of age or older; (6) they had complete medical records; and (7) they agreed to participate in the study. All the subjects underwent a diagnostic interview with one of the authors (MATU or EJP) who were experienced in management of headache cases, to make decisions on inclusion in the study. Participants with EM and CM were referenced in the same way in all centers, as a precaution to reduce selection bias resulting from differences in the levels of complexity of cases handled at these centers. We excluded subjects who: (1) withdrew the consent statement; or (2) developed a new headache in the interval between the invitation and the study interview. Interviews were conducted through using a semi-structured questionnaire and took place between August 2018 and January 2020.

Body mass index (BMI) was calculated using the subjects’ self-reported weight and height. Cardiovascular risk factors were recorded as binary variables that indicated the presence of at least one of the following: hypertension, diabetes, dyslipidemia, cardiovascular or cerebrovascular disease. Regular amounts of cigarette and illicit drug consumption were recorded as three-level categorical variables: never, past and current. Presence of alcohol consumption on a weekly basis was recorded as a binary variable (i.e. present or absent). The adequacy of aerobic physical activity was recorded in accordance with the World Health Organization recommendations: ≥150 minutes (moderate intensity) or ≥75 minutes (vigorous intensity) per week. The monthly household income per resident was calculated to evaluate possible effects from the socioeconomic class. The monthly income of all working residents was added up and divided by the total number of residents (either active workers or not).

The number of years with migraine was taken to be the period between the onset of typical migraine symptoms and the interview date. As mentioned earlier, only individuals whose migraine had been present for at least six months were included. The pain intensity was classified as mild (no limitation), moderate or severe (disabling). Pain that was described as pulsatile and/or pressing was recorded as a four-level variable: never, occasionally, most times and always. Associated symptoms during migraine attacks were recorded as binary variables and these included nausea, vomiting, photophobia, phonophobia and movement and tactile allodynia. Movement allodynia was considered to exist if the headache was aggravated through routine physical activity. Headache frequency (days/month) was calculated as the average over the last six months in order to better consider fluctuations in the frequency of attacks^9^.

**Figure 1:**
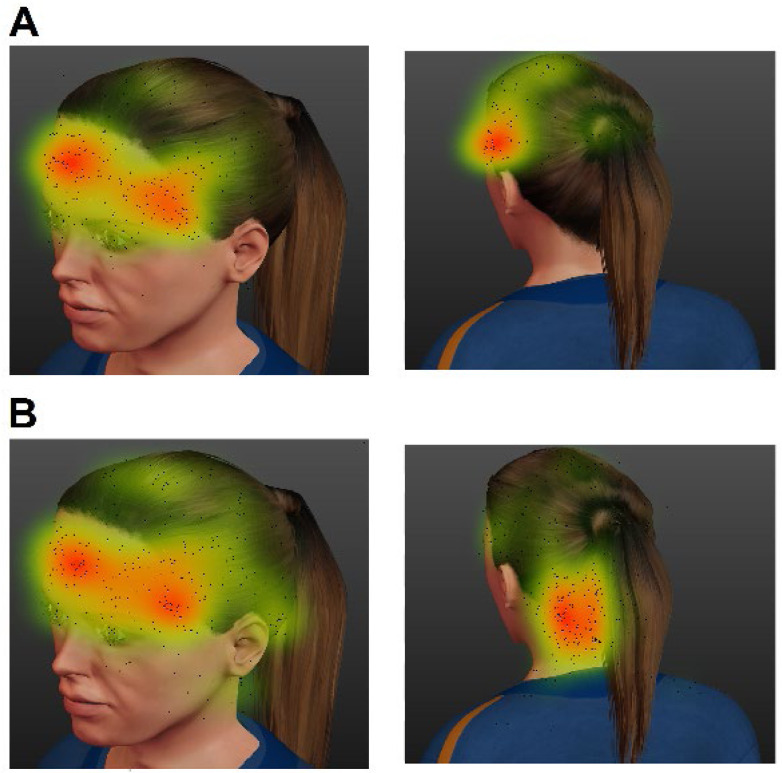
(A) Migraineurs with pain restricted to the trigeminal areas (non-convergent pain group). (B) Migraineurs with pain beyond the trigeminal area (convergent pain group). Each dark point represents a pain location selected by a patient. This heat map scale uses a Kernel density estimate that translates the proportion of points in an area into a color scale. Therefore, the scales are not the same for all images. However, all color scales vary from green to red, with the latter indicating a higher point density.

Each subject was asked if the attacks were: (1) predominantly on the right side; (2) predominantly on the left side; (3) predominantly unilateral, but with possible side shifts during attacks; or (4) predominantly bilateral. The patients were then asked to select the locations that represented the most prevalent areas involved in their usual attacks, through mouse clicks on an electronic form. Two images representing the anterolateral and posterolateral views of a model head (Figure 1) were presented to each participant, who could indicate up to three points in each image. The coordinates of each point were automatically classified according to 20 different head/neck regions: frontal, temporal, parietal, supraorbital, orbital, infraorbital, nasal, occipital, suboccipital, anterior cervical, sternocleidomastoid, lateral cervical, posterior cervical, zygomatic, auricular, mastoid, oral, mental, buccal and parotid^10^. We classified subjects as having extratrigeminal pain (convergent group) if they selected a site outside of the trigeminal regions (occipital, suboccipital, sternocleidomastoid and anterior/lateral/posterior cervical). Otherwise, the participants were included in the non-convergent group. Areas with mixed innervation (e.g. auricular and temporal sites) were considered to be trigeminal areas.

Each subject was asked about the presence of 44 symptoms in a typical migraine attack. All the symptoms were presented in a random order and were classified into six domains based, in part, on the neuroanatomic-correlated classification presented by Karsan^11^: (1) behavioral/cognitive impairment: hyperactivity, dyscognition, inattention, fatigue, depression symptoms and irritability; (2) homeostatic changes: constipation, urinary urgency, hyperphagia, polydipsia, hypertension, hypotension, diarrhea, yawning, pallor and hyporexia; (3) non-painful migrainous symptoms/altered sensory sensitivity: nausea, vomiting, photophobia, phonophobia, osmophobia, tactile allodynia and nuchal rigidity; (4) cortical aura-like symptoms: negative and positive visual symptoms, negative and positive sensory symptoms and fluent and non-fluent dysphasia; (5) brainstem aura-like symptoms: dysarthria, vertigo, tinnitus, hypoacusis, diplopia, ataxia and decreased level of consciousness; and (6) cranial autonomic changes: conjunctival injection, lacrimation, nasal congestion, rhinorrhea, eyelid edema, facial sweating, miosis and ptosis. The presence of tactile allodynia was based on the first question of the Brazilian version of the 12-item Allodynia Symptom Checklist^12^.

Presence of medication overuse and prodromic nuchal rigidity (within the 72 hours prior to the headache) was recorded as binary variables. Use of preventive pharmacological treatment for migraine was classified as never, past or current. The impact of the headache was assessed using the Migraine Disability Assessment (MIDAS)^13^. Symptoms of depression and anxiety were quantified using the Patient Health Questionnaire-9 (PHQ-9) and the 7-item Generalized Anxiety Disorder Questionnaire (GAD-7) scales, respectively^14,15^.

All statistical analyses were conducted using R version 4.0.2. The Shapiro-Wilk test and quantile-quantile plots were used to check for normality. Accordingly, the sample data were summarized as mean±standard deviation, median (interquartile range) and count (percentage proportion). For visual exploration of the distribution of pain sites, kernel estimation was used to analyze the density of points in the two-dimensional plane. Missed data were dealt with through imputation via a k-nearest neighbor algorithm. To analyze differences among the groups, the one-way ANOVA test for numerical variables (Kruskal-Wallis test when the assumptions of ANOVA were not met) or the chi-square test for categorical variables (Fisher test when the expected count in any cell was less than five) was used. For post-hoc analysis, the Tukey honest significant difference or Dunn test adjusted with the Holm method were used for numerical variables. In the case of categorical variables, regression modeling adjusted through the Tukey method was used. A multivariate logistic regression model was fitted with the presence of convergence as the dependent variable. This model was selected using a backward elimination algorithm and the corrected Akaike information criterion. To assess the model fit, we used the following: residual analysis, ratio of residual deviance to residual degrees of freedom, Hosmer and Lemeshow test, Osius-Rojek test, Stukel test and influence analysis. All tests were performed at a significance level of 0.05. No sample size calculation was conducted a priori for this secondary data analysis.

## RESULTS

We invited a total of 254 subjects to participate in the study, of whom 212 agreed to this (83%). After the study interview, we decided to exclude two patients. One of them experienced a new headache that resembled an episodic paroxysmal hemicrania and the other patient experienced a new cervicogenic headache. Both of them suffered from CM. A total of 97 patients (46%) were diagnosed with EM and 113 (54%) with CM. The former group consisted of 76 cases of migraine without aura (78%) and 21 cases of migraine with aura (22%). In the CM group, 78 patients (69%) overused medications to alleviate their condition. The subjects’ mean age was 39.45±12.63 years and 189 (90%) were female.

The general sample characteristics are shown in Table 1. Convergence was part of the manifestation of 116 cases (55%). To investigate the variables potentially associated with occurrence of convergent pain, independently of chronification status, we stratified the sample according to the presence of convergence and chronification. There were significant differences in monthly household income, presence of cardiovascular risks and presence of weekly alcohol consumption. For the first two variables, the difference was only significant when comparing the two groups characterized by being the least and the most severe groups, i.e. EM in the non-convergent group and CM in the convergent group (monthly household income: p=0.01 and cardiovascular risk: p=0.02). However, in the stratum defined by the non-convergent group, the prevalence of weekly alcohol consumption was higher for EM than for CM (p=0.021).

**Table 1:** General features stratified according to the presence of convergence and chronification.

		Episodic migraine (n=97)		Chronic migraine (n=113)		p-value* †	Post-hoc p-values* ‡					
		Non-convergent (n=48)	Convergent (n=49)	Non-convergent (n=46)	Convergent (n=67)		EMNC vs. EMC	EMNC vs. CMNC	EMNC vs. CMC	EMC vs. CMNC	EMC vs. CMC	CMNC vs. CMC
**Sociodemographic variables**												
	Age (years)	37.1±13.6	39.3±11.3	39.6±13.4	41.1±12.0	0.422						
	Skin color: white	42 (87.5%)	37 (75.5%)	34 (73.9%)	49 (73.1%)	0.275						
	Gender: female	42 (87.5%)	46 (93.9%)	40 (87.0%)	61 (91.0%)	0.608						
	Marital status: married	23 (47.9%)	33 (67.3%)	32 (69.6%)	39 (58.2%)	0.118						
**Income and occupation**												
	Employed	33 (68.8%)	33 (67.3%)	24 (52.2%)	39 (58.2%)	0.288						
	Monthly household income per resident (Brazilian real)	2500.0 (2165.0)	2000.0 (2633.0)	1583.0 (1942.0)	1600.0 (1500.0)	**0.014**	0.412	0.121	**0.010**	0.851	0.468	0.494
**Cardiovascular risk factors and lifestyle**												
	BMI (kg/m^2^)	24.1 (7.3)	25.7 (5.9)	25.6 (6.7)	26.1 (7.6)	0.172						
	Cardiovascular risk: present§	13 (27.1%)	20 (40.8%)	18 (39.1%)	36 (53.7%)	**0.039**	0.478	0.598	**0.020**	0.998	0.512	0.418
	Current or former smoker	8 (16.7%)	10 (20.4%)	12 (26.1%)	19 (28.4%)	0.463						
	Weekly alcohol consumption	17 (35.4%)	7 (14.3%)	5 (10.9%)	9 (13.4%)	**0.005**	0.069	**0.021**	**0.028**	0.958	0.999	0.977
	Adequate physical activity¶	11 (22.9%)	9 (18.4%)	10 (21.7%)	9 (13.4%)	0.557						

EMNC: episodic migraine with non-convergent pain; EMC: episodic migraine with convergent pain; CMNC: chronic migraine with non-convergent pain; CMC: chronic migraine with convergent pain; BMI: body mass index. All data are summarized as mean±standard deviation, median (interquartile ratio), or count (frequency, %) according to the variable type and distribution.

* p-values<0.05 are indicated in bold.

† One-way ANOVA (or Kruskal-Wallis) test for numerical variables or chi-square (or Fisher) test for categorical variables.

‡ Tukey honest significant difference (or Dunn) test for numerical variables or regression modeling for categorical variables.

§ At least one of the following: hypertension, diabetes, dyslipidemia, cardiovascular events and neurovascular events.

¶ At least 150 minutes of moderate-intensity or 75 minutes of vigorous-intensity aerobic physical activity.

The pain location mapping is shown in Figure 1. The point density was higher in the posterior cervical region for the convergent group. There was no significant difference for any trigeminal site between the convergent and non-convergent groups (Fisher test; p>0.05). The posterior cervical site was more often involved in the chronic than in the episodic migraine group (33 vs. 21%; p=0.034).

Only three subjects had had less than a year of migraine (all were cases of EM beginning in the last six months). Most participants in the EM group (90.7%) and CM group (79.6%) reported having a relatively stable attack frequency, with averages of less than 14 and more than 14 headache days in the last six months, respectively. The individuals in the CM group with a lower frequency of headache had a more fluctuating course (n=19; 10–14 headache days), as did the patients with EM who had only recently evolved with chronification (n=4; <10 headache days). The subjects with EM who reported having an average of more than 14 headache days (n=9) were the ones who might evolve to CM if they were followed up longitudinally. None of these nine cases presented less than six months of migraine (median 10.3 years; range 3.5–33.5 years). The headache characteristics are shown in Table 2. There were significant differences (p<0.001) in migraine severity, headache frequency, use of preventive drugs and presence of medication overuse. However, most of the differences were due to the chronification status.

**Table 2:** Headache features stratified according to the presence of convergence and chronification.

	Episodic migraine (n=97)		Chronic migraine (n=113)		p-value* †	Post-hoc p-values* ‡					
	Non-convergent (n=48)	Convergent (n=49)	Non-convergent (n=46)	Convergent (n=67)		EMNC vs. EMC	EMNC vs. CMNC	EMNC vs. CMC	EMC vs. CMNC	EMC vs. CMC	CMNC vs. CMC
MIDAS score	18.0 (33.5)	25.0 (48.0)	52.5 (49.5)	71.0 (83.0)	**<0.001**	0.266	**0.012**	**<0.001**	0.356	**0.012**	0.218
Migraine duration (years)	8.5 (11.2)	10.0 (19.0)	10.0 (16.4)	13.0 (19.8)	0.080						
Pain side: fixed-side	13 (27.1%)	16 (32.7%)	15 (32.6%)	19 (28.4%)	0.154						
Pain side: unilateral with side-shifts	20 (41.7%)	11 (22.4%)	8 (17.4%)	24 (35.8%)							
Pain side: bilateral	15 (31.3%)	22 (44.9%)	23 (50.0%)	24 (35.8%)							
Headache days per month: <10	33 (68.8%)	31 (63.3%)	3 (6.5%)	1 (1.5%)	**<0.001**	Ref.	Ref.	Ref.	Ref.	Ref.	Ref.
Headache days per month: 10-14	11 (22.9%)	13 (26.5%)	7 (15.2%)	12 (17.9%)		0.633	**0.012**	**0.001**	**0.025**	**0.002**	0.190
Headache days per month: >14	4 (8.3%)	5 (10.2%)	36 (78.3%)	54 (80.6%)		0.699	**<0.001**	**<0.001**	**<0.001**	**<0.001**	0.201
Use of preventive drug: never	27 (56.3%)	27 (55.1%)	15 (32.6%)	14 (20.9%)	**<0.001**	Ref.	Ref.	Ref.	Ref.	Ref.	Ref.
Use of preventive drug: past	9 (18.8%)	8 (16.3%)	8 (17.4%)	17 (25.4%)		0.833	0.420	**0.014**	0.323	**0.009**	0.147
Use of preventive drug: current	12 (25.0%)	14 (28.6%)	23 (50.0%)	36 (53.7%)		0.747	**0.010**	**<0.001**	**0.020**	**<0.001**	0.258
Medication overuse	10 (20.8%)	14 (28.6%)	31 (67.4%)	47 (70.1%)	**<0.001**	0.812	**<0.001**	**<0.001**	**0.001**	**<0.001**	0.990

EMNC: episodic migraine with non-convergent pain; EMC: episodic migraine with convergent pain; CMNC: chronic migraine with non-convergent pain; CMC: chronic migraine with convergent pain; MIDAS: Migraine Disability Assessment test. All data are summarized as mean±standard deviation, median (interquartile ratio), or count (frequency, %) according to the variable type and distribution.

* p-values<0.05 are indicated in bold.

† One-way ANOVA (or Kruskal-Wallis) test for numerical variables or chi-square (or Fisher) test for categorical variables.

‡ Tukey honest significant difference (or Dunn) test for numerical variables or regression modeling for categorical variables. Ref.: reference level.

The results regarding PHQ-9 and GAD-7 scores and the distribution of the associated symptoms during migraine attacks are shown in Table 3. There were significant differences for all the variables except for the cortical aura-like symptom group (p=0.094). However, most of these differences were due to the chronification status. Interestingly, however, patients with convergent pain showed more migrainous/altered sensitivity symptoms in the EM group (p=0.025) and CM group (p=0.002). Also, those with convergent pain showed more symptoms typical of brainstem aura in the EM stratum (p=0.007).

**Table 3:** PHQ-9 and GAD-7 scores and number of attack symptoms per group stratified according to the presence of convergence and chronification.

	Episodic migraine (n=97)		Chronic migraine (n=113)		p-value* †	Post-hoc p-values* ‡					
	Non-convergent (n=48)	Convergent (n=49)	Non-convergent (n=46)	Convergent (n=67)		EMNC vs. EMC	EMNC vs. MNC	EMNC vs. CMC	EMC vs. CMNC	EMC vs. CMC	CMNC vs. MC
GAD-7 (anxiety)	8.0 (7.3)	7.0 (9.0)	12.0 (8.0)	11.0 (8.5)	**0.037**	0.762	**0.030**	0.193	0.266	0.778	0.737
PHQ-9 (depression)	6.0 (7.0)	8.0 (8.0)	9.5 (8.8)	10.0 (7.0)	**0.015**	0.601	**0.020**	0.055	0.371	0.478	0.518
Migrainous symptoms and/or altered sensory sensitivity	4.0 (2.0)	5.0 (2.0)	4.0 (2.0)	6.0 (2.0)	**<0.001**	**0.025**	0.998	**0.003**	**0.015**	0.968	**0.002**
Behavior/cognitive symptoms	3.0 (2.0)	4.0 (3.0)	4.0 (2.8)	4.0 (2.0)	**0.008**	0.371	0.123	**0.004**	0.461	0.331	0.723
Cortical aura-like symptoms	1.0 (2.3)	1.0 (4.0)	1.0 (3.8)	2.0 (3.0)	0.094						
Brainstem aura-like symptoms	1.0 (2.0)	2.0 (4.0)	1.0 (2.8)	2.0 (3.0)	**0.001**	**0.007**	0.314	**0.002**	0.444	0.998	0.288
Homeostatic symptoms	1.0 (3.0)	2.0 (3.0)	2.0 (3.0)	2.0 (3.5)	**0.014**	0.236	0.321	**0.006**	0.999	0.573	0.487
Cranial autonomic symptoms	1.0 (2.0)	1.0 (2.0)	1.0 (2.0)	2.0 (4.0)	**0.038**	0.274	0.852	**0.044**	0.814	0.532	0.292

EMNC: episodic migraine with non-convergent pain; EMC: episodic migraine with convergent pain; CMNC: chronic migraine with non-convergent pain; CMC: chronic migraine with convergent pain; GAD-7: 7-item Generalized Anxiety Disorder Questionnaire; PHQ-9: Patient Health Questionnaire-9. All data are summarized as mean±standard deviation, median (interquartile ratio), or count (frequency, %) according to the variable type and distribution.

* p-values<0.05 are indicated in bold.

† One-way ANOVA (or Kruskal-Wallis) test for numerical variables or chi-square (or Fisher) test for categorical variables.

‡ Tukey honest significant difference (or Dunn) test for numerical variables or regression modeling for categorical variables.

The model selected was adjusted for the chronification status, PHQ-9 score, BMI, MIDAS score and migrainous/altered sensitivity and brainstem aura-like symptom groups. There was a significant difference regarding the presence of one migrainous/altered sensitivity symptom (OR=1.39; 95%CI 1.14–1.71) and marginal evidence for an increase in MIDAS score of 10 points (OR=1.06; 95%CI 0.99–1.13). The group characterized by migrainous/altered sensitivity symptoms was a composite one. Further analysis on each symptom found that there were statistical associations between convergent pain and vomiting (p=0.045), tactile allodynia (p<0.001), nuchal rigidity (p<0.001) and movement allodynia (p=0.031).

## DISCUSSION

Extratrigeminal pain in migraine, which is an expression of the convergence phenomenon, was characterized by involvement of the posterior cervical site or neck pain (NP). Presence of convergence was associated with occurrence of migrainous/altered sensitivity symptoms, mainly represented by allodynia. There was a trend towards an association between convergence and migraine severity, although it was not statistically significant at an alpha level of 0.05. These findings were independent of the chronification status. However, given that allodynia and increased migraine severity are typically found in CM, it is not surprising that we found convergence in this group more often.

In migraineurs, this convergence is expressed as an association of pain in the area supplied by the trigeminal nerve and in the first cervical roots (C1 and C2) during an attack. Usually, cervical pain or discomfort arises in the C1/C2 dermatome during the premonitory phase, while trigeminal pain appears hours or days (up to 72 hours) after the cervical symptoms^16^.

The intriguing involvement of the neck in migraine pain has long been recognized^17^. Compared with our findings, most studies have reported higher neck pain (NP) prevalence in migraine. A study conducted in a headache clinic showed NP prevalence of 70.5%^18^. In a population study, NP prevalence was 76.2% among those with pure migraine, 89.3% among those with coexisting tension-type headache (TTH) and 83.3% among those with episodic migraine with or without episodic TTH^19^. Our recording method and the three-point limitation per image might have been responsible for this discrepancy.

Interestingly, the abovementioned population study also showed that the frequency of migraine attacks is correlated with the number of days with NP^19^. The largest study dedicated to this topic reported that there was higher frequency of NP with migraine chronification, along with higher frequency of diffuse occipital pain^2^. In a prospective study on a sample selected in both a headache clinic and the general community, NP prevalence varied according to headache pain intensity: mild (42.8%), moderate (61.1%) and severe (72.6%)^20^. Recent use of pain drawings has shown marginal evidence for greater pain extent in the posterior region of the head in the CM group, compared with the EM group^4^. Therefore, associations of frequent migraine attacks and/or CM with the presence of NP have been a recurrent finding in different studies. Those findings corroborate ours, thus suggesting that patients with CM have higher rates of trigeminocervical convergence.

In addition to this association with group classification, NP was also associated with the following: presence of at least one cardiovascular risk factor; longer-term migraine; more diffuse, frequent and intense attacks; presence of mechanical and tactile allodynia; presence of medication overuse; and prodromal nuchal rigidity. Presence of more diffuse pain was the most important NP-associated factor. NP could simply represent a preferred location in the pain spreading process that is seen in individuals with higher chronification risk. Data from the Chronic Migraine Epidemiology and Outcomes Study (CaMEO) were used to investigate associations between the presence of non-cephalic pain in eight body regions and occurrence of EM-to-CM progression and CM persistence over three months^21^. At the baseline, the CM group showed 1.09-1.29 times more non-cephalic pain locations than did the EM group. At three months, each additional location exerted some effect on CM odds independently of other covariates (demographics, depression/anxiety, allodynia, BMI and baseline acute headache treatment).

Calhoun et al. explored the role of NP in migraineurs through a series of studies, and they found that: (1) NP was prevalent in migraine; (2) its presence on the day preceding migraine was associated with treatment resistance; and (3) it was a predictor of disability, independent of migraine frequency and severity^22^. They raised the possibility that NP in migraineurs represents hyperalgesia or allodynia. This was in line with our findings that the presence of migrainous/altered sensitivity symptoms is associated with convergence, independently of the chronification status.

Although we did not explore pressure sensitivity, several studies have demonstrated lower pressure-pain thresholds (PPTs) in migraineurs, compared with controls, including in the cervical and distant extra-trigeminal areas^23–25^. An anterior-to-posterior PPT gradient^23^ in the scalp of migraineurs (and healthy controls) was found, mimicking the sites discussed above as the ones most frequently affected by migraine pain. However, the method used in that study differed essentially from the one used in the present study because it focused on objective measurement of static mechanical pain hyperalgesia. By asking migraineurs to indicate or draw the sites most frequently affected by pain, we produced an alternative, meaningful and quick way to explore the anatomical features of the complaint.

To the best of our knowledge, this was the largest study to use an explicit non-verbal recording method to locate migraine pain. The diagnosis and evaluation by a headache specialist further supported our findings, as did our use of balanced groups of individuals with EM and CM. Our study included patients treated in different settings and had a participation rate of 83%. The general characteristics of our sample were comparable with those in other studies carried out in headache centers. Therefore, our results seem to apply to CM and EM patients treated at these headache units.

Nonetheless, some limitations need to be considered, namely: (1) the non-longitudinal design allowed us to establish associations of some explanatory variables with NP that were not as causal relationships; (2) we did not stipulate any migraine-free period before the interview, and memory bias may have interfered with our results; (3) we did not use any standardized instrument to measure allodynia; (4) as discussed earlier, the six-point limitation may have caused underestimation of the number of sites affected by migraine pain; and (5) our results were mainly based on patient reports and medical records.

Future studies should consider a prospective record of pain location using pain drawings or registering more points per attack. Also, recording whether the neck and other subregions respond differently to established migraine treatments would be interesting.

In conclusion, while migraine attacks most commonly involve the frontotemporal regions, the convergence phenomenon is more common in chronic migraineurs. Some commonly observed features such as tactile allodynia and greater severity of disease are associated with this extratrigeminal site of pain.

## References

[1] (2018). Cephalalgia.

[2] Kelman L (2005). Migraine pain location: a tertiary care study of 1283 migraineurs. Headache.

[3] Fernández-de-Las-Peñas C, Falla D, Palacios-Ceña M, Fuensalida-Novo S, Arias-Buría JL, Schneebeli A (2018). Perceived pain extent is not associated with widespread pressure pain sensitivity, clinical features, related disability, anxiety, or depression in women with episodic migraine. Clin J Pain.

[4] Uthaikhup S, Barbero M, Falla D, Sremakaew M, Tanrprawate S, Nudsasarn A (2020). Profiling the extent and location of pain in migraine and cervicogenic headache: a cross-sectional single-site observational study. Pain Med.

[5] Piovesan EJ, Kowacs PA, Oshinsky ML (2003). Convergence of cervical and trigeminal sensory afferents. Curr Pain Headache Rep.

[6] Piovesan EJ, Kowacs PA, Tatsui CE, Lange MC, Ribas LC, Werneck LC (2001). Referred pain after painful stimulation of the greater occipital nerve in humans: evidence of convergence of cervical afferences on trigeminal nuclei. Cephalalgia.

[7] Dilli E, Halker R, Vargas B, Hentz J, Radam T, Rogers R (2015). Occipital nerve block for the short-term preventive treatment of migraine: a randomized, double-blinded, placebo-controlled study. Cephalalgia.

[8] Andreou AP, Edvinsson L (2019). Mechanisms of migraine as a chronic evolutive condition. J Headache Pain.

[9] Serrano D, Lipton RB, Scher AI, Reed ML, Stewart WBF, Adams AM (2017). Fluctuations in episodic and chronic migraine status over the course of 1 year: implications for diagnosis, treatment and clinical trial design. J Headache Pain.

[10] Moore KL, Dalley AF, Agur AMR (2010). Clinically Oriented Anatomy.

[11] Karsan N, Bose P, Goadsby PJ (2018). The Migraine Premonitory Phase.

[12] Florencio LL, Chaves TC, Branisso LB, Gonçalves MC, Dach F, Speciali JG (2012). 12 item allodynia symptom checklist/Brasil: cross-cultural adaptation, internal consistency and reproducibility. Arq Neuro-Psiquiatr.

[13] Fragoso YD (2002). MIDAS (Migraine Disability Assessment): a valuable tool for work-site identification of migraine in workers in Brazil. Sao Paulo Med J.

[14] Kroenke K, Spitzer RL, Williams JB (2001). The PHQ-9: validity of a brief depression severity measure. J Gen Intern Med.

[15] Spitzer RL, Kroenke K, Williams JBW, Löwe B (2006). A brief measure for assessing generalized anxiety disorder: the GAD-7. Arch Intern Med.

[16] Giffin NJ, Ruggiero L, Lipton RB, Silberstein SD, Tvedskov JF, Olesen J (2003). Premonitory symptoms in migraine: an electronic diary study. Neurology.

[17] Blau JN, MacGregor EA (1994). Migraine and the neck. Headache.

[18] de Queiroz LP, Rapoport AM, Sheftell FD (1998). Clinical characteristics of migraine without aura. Arq Neuro-Psiquiatr.

[19] Ashina S, Bendtsen L, Lyngberg AC, Lipton RB, Hajiyeva N, Jensen R (2015). Prevalence of neck pain in migraine and tension-type headache: a population study. Cephalalgia.

[20] Calhoun AH, Ford S, Millen C, Finkel AG, Truong Y, Nie Y (2010). The prevalence of neck pain in migraine. Headache.

[21] Lipton RB, Fanning KM, Buse DC, Martin VT, Reed ML, Manack Adams A (2018). Identifying natural subgroups of migraine based on comorbidity and concomitant condition profiles: results of the Chronic Migraine Epidemiology and Outcomes (CaMEO) study. Headache.

[22] Calhoun AH, Ford S, Pruitt AP (2011). Presence of neck pain may delay migraine treatment. Postgrad Med.

[23] Barón J, Ruiz M, Palacios-Ceña M, Madeleine P, Guerrero ÁL, Arendt-Nielsen L (2017). Differences in topographical pressure pain sensitivity maps of the scalp between patients with migraine and healthy controls. Headache.

[24] Palacios-Ceña M, Lima Florencio L, Natália Ferracini G, Barón J, Guerrero ÁL, Ordás-Bandera C (2016). Women with chronic and episodic migraine exhibit similar widespread pressure pain sensitivity. Pain Med.

[25] Florencio LL, Giantomassi MCM, Carvalho GF, Gonçalves MC, Dach F, Fernández-de-Las-Peñas C (2015). Generalized pressure pain hypersensitivity in the cervical muscles in women with migraine. Pain Med.

